# Identification and Evaluation of Hazardous Pyrolysates in Bio-Based Rigid Polyurethane-Polyisocyanurate Foam Smoke

**DOI:** 10.3390/polym13193205

**Published:** 2021-09-22

**Authors:** Sanita Reinerte, Vilhelmine Jurkjane, Ugis Cabulis, Arturs Viksna

**Affiliations:** 1 Faculty of Chemistry, University of Latvia, LV-1004 Riga, Latvia; arturs.viksna@lu.lv; 2 Latvian State Institute of Wood Chemistry, LV-1006 Riga, Latvia; ligms@edi.lv (V.J.); ugis.cabulis@kki.lv (U.C.)

**Keywords:** rigid PU-PIR foam, analytical pyrolysis, physical hazards, health hazards, environmental hazards

## Abstract

In this study, rigid polyurethane (PU) and polyisocyanurate (PIR) foam samples made from renewable material (tall oil fatty acid) based polyols were analyzed by pyrolysis gas chromatography mass spectrometry (Py-GC/MS) to obtain information about the full relative smoke content, with a focus on substance identification by their functional groups and hazardousness. The relative content of gaseous products produced during the thermal degradation was evaluated between the two samples, differenced by their assigned isocyanate (NCO) index value—150 and 300. The main thermal degradation components of the rigid PU-PIR foam were found to originate from the decomposition of isocyanate, primarily forming 4,4′-methylenedianiline, 3,3′-diaminodiphenylmethane, N-methylaniline, aniline, 4-benzylaniline and phenyl isocyanate. Hazard analysis revealed that the most common hazards were the hazards related to health: H315 (36%), H319 (28%), H335 (25%), and H302 (23%). The chemical compound with the highest relative content value—4,4′-methylenedianiline (45.3% for PU and 52.4% for PIR)—was identified to be a suspected carcinogen and mutagen. The focus of the study was identifying and evaluating the relative quantities of the produced gaseous products, examine their hazardousness, and provide information on the released thermal degradation products to form a renewable-source based rigid PU and PIR foam.

## 1. Introduction

For more than a decade, bio-based rigid polyurethane (PU) and polyisocyanurate (PIR) foams have been investigated as promising building thermal insulation materials. With technological parameters often equal to or even better than those of conventionally produced foams [1,2,3,4,5,6,7,8,9,10], they are regarded as an environmentally safer alternative to the conventional oil-based rigid PU and PIR materials available in the market.

Although one of the two basic ingredients for producing rigid PU-PIR foams—polyols—can be fully sustainable when produced from renewable sources like vegetable oils and lignin [11,12,13,14], the other part—isocyanates (NCO)—are more difficult to find alternatives for. Therefore, NCO-based rigid PU-PIR foams are still the dominant PU-PIR foams in the PU industry [15]. The NCO prevalence in a thermal insulation material meant for public buildings and homes is a factor that must be acknowledged if an event like a house-fire occurs. The fumes excreted in fires where NCO-based materials are present are a great concern for the environment, and individual and public health. Their released fire gases are made of fully and partially oxidized products, as well as fuel and its degradation products [16]. Using the steady state tube furnace, Stec and Hull assessed the fire toxicity of building insulation materials, concluding that out of the six studied materials, PU and PIR foams produced the most of the toxic products [17].

Thermal decomposition of the PU itself is usually a reverse of polymerization, resulting in the formation of their precursor functional compounds—diisocyanates, diamines and dihydroxy compounds—and the products of decomposition can be predicted from the composition of the polymer [18]. However, these observations stem from the studies investigating conventional material-based PU. There is limited information about the thermal decomposition products from renewable source-based PU materials and their hazards. Thus far, the hazard reducing effects of smoke suppressants in the PU foam have been studied, either as solid inorganic additives [19,20,21] or aerogel composites [22,23]. Hiltz has determined that an analytical pyrolysis method consisting of a pyrolysis gas chromatograph coupled with a mass spectrometer (Py-GC/MS) can be used to differentiate the thermal degradation products of various polyether urethanes, polyureas and polyurethane ureas, and provide information on the materials used in their preparation [24]. This is a Py-GC/MS method application to determine material quality by analyzing its thermal degradation product composition and product relative content.

Hypothetically, due to the branched and long-chain structures from the fatty-acid based polyols (MW > 1000 g/mol) the polyols produced from bio-based sources could affect the overall thermal decomposition gaseous product chemical composition, producing different chemical products every time. Conventional polyols are normally short-chain polyether or polyester polyols (MW < 1000 g/mol), where the molecules tend to cleave through a predetermined pyrolysis mechanism, forming specific chemical compounds. Therefore, the present work is a comparative study where both the rigid PU and PIR foam samples were made from a bio-based material—a high-functionality tall-oil fatty acid polyol—in order to assess the differences in the thermal degradation gaseous compound composition and distribution between the rigid PU foam (NCO 150) and the rigid PIR foam (NCO 300). The produced pyrolytic products were fully identified for both the rigid PU foam and the rigid PIR foam. The identified pyrolytic products were evaluated from both their functional group distribution and the hazards identified with them using the hazard statements of the Globally Harmonized System of Classification and Labelling of Chemicals (GHS) [25].

## 2. Materials and Methods

The samples analyzed in this research have been studied earlier using thermogravimetric analysis [10], which, along with the obtained thermal degradation behavior data, allowed quantifying the volatile product and solid residue of the rigid PU-PIR foam samples. Additionally, these samples have been used in the assessment of gas excretion trends for specific chemical compounds by employing a differential thermal analysis method coupled with Fourier transform infrared spectrometry [26].

### 2.1. Materials

The rigid PU-PIR foam formulations were made by utilizing the chemicals presented in Table 1. The tall oil fatty acid (TOFA)-based polyol was produced by epoxidating the TOFAs (Forchem Oyj, Rauma, Finland), opening the introduced oxirane rings and esterificating them with a polyfunctional alcohol (trimethylolpropane). The full description of the synthesis method for the TOFA-based polyol is presented in this paper [27].

### 2.2. Methodology

The TOFA polyol-based PU-PIR formulations were designed according to their NCO indexes 150 and 300 (Table 2). The necessary components were weighted and stirred into a homogeneous mixture (1 min/2000 rpm). Afterwards, the polyol formulation mixture was de-gassed for no less than 2 h (T_room_).

Once de-gassed, the polyol components and the respective NCO moieties were weighed and mixed (15 s/2000 rpm). The PU-PIR formulation was poured into an open top mold and cured in a curing oven (50 °C/2 h).

A Frontier Lab (Fukushima, Japan) free-fall mechanism Micro Double-shot Pyrolyzer Py-2020iD (Tpyrolysis 650 °C; v 650 °C/s), directly coupled with a Shimadzu GC/MS-QP 2010 apparatus (Shimadzu, Kyoto, Japan) (60 m × 0.25 mm × 0.25 mm capillary column RTX-1701 (Restec, Metairie, LA, USA)) was used to perform Py-GC/MS (T_injector_ 250 °C; EI mode (70 eV); MS scan range 15–350 m/z; Q_sample_ (He) 1 mL/min; split ratio 1:15). Oven program: isothermal at 60 °C/1 min, ramp rate 7 °C/min to 270 °C, final hold 270 °C for 15 min. Library MS NIST 147.LI13 was used for identifying individual compounds. The summed molar areas of the relevant peaks were normalized to 100%, and the data of repetitive pyrolysis experiments were averaged. The relative error of measurements was 1–3%. The results were from triplicate experiments (m_sample_ = 1.00 mg).

## 3. Results and Discussion

Overall, the Py-GC/MS results revealed that the main difference between the sample pyrolysates were the changes in the individual chemical compound output content, but not the hypothesized changes in the product chemical composition.

For a clearer understanding of the produced chemical component quantitative composition, the characteristics measured for F_HF95 NCO 150 and F_HF95 NCO 300 are referenced in Table 3 from one of the previous studies on the thermal degradation process analysis for these formulations [10].

The Fourier transform infrared spectral analysis (FTIR) data on the material composition are also available in the aforementioned study [10], but the thermogravimetric, differential thermal analysis and the FTIR data on the released gaseous compounds from these formulations are available in this paper [26].

### 3.1. Py-GC/MS Gaseous Product Characterization

**Figure 1:**
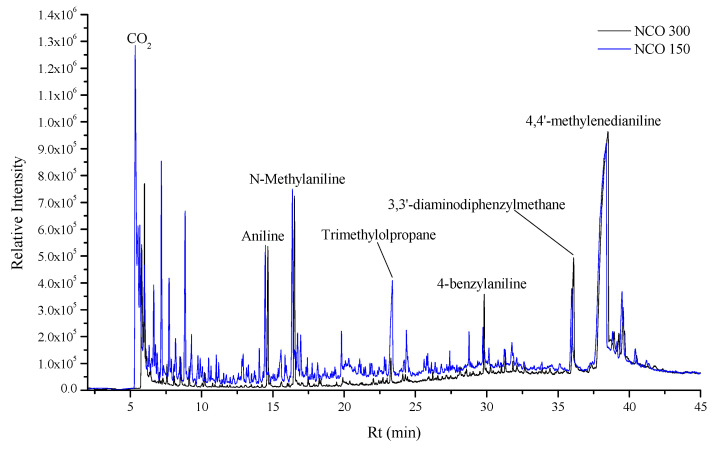
Py-GC/MS chromatograms obtained for F_HF95 samples.

**Figure 2:**
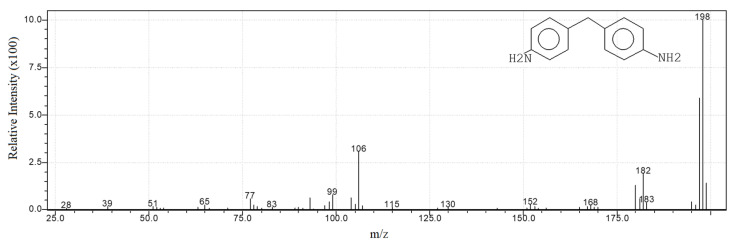
Mass spectrum of 4,4′-methylenedianiline.

The obtained Py-GC/MS curves of the two samples are shown in Figure 1 in an overlaid form to perceive the relative intensity differences better, while Figure 2 as an example of the obtained mass spectra shows the mass spectrum of the main thermal decomposition product 4,4′-methylenedianiline. Py-GC/MS provides chromatograms with information on the thermal degradation product distribution based on their retention time (Rt) and chemical information on the thermal decomposition products of PUs through their mass spectra.

The full list of the identified chemical compounds and GHS hazard statements is available in Table 4, the GHS hazard statements according to the Commission Regulation (EU) 2018/669 [25]. The values expressed are the relative quantities (%) for the chemical compounds out of the full obtained pyrolytic compound data, measured by the Shimadzu GC/MS-QP 2010 data processing program.

Quantity change Δ (%) was calculated using Equation (1):

(1) $$∆\left(\%\right)=m_{\mathrm{rel}}\mathrm{NCO}\;300\;\left(\%\right)-{\;m}_{\mathrm{rel}}\mathrm{NCO}\;150\;\left(\%\right)$$

where m_rel_ F_HF95 NCO 150 (%) is the percentual value of a chosen chemical compound quantity for F_HF95 NCO 150 formulation, m_rel_ F_HF95 NCO 300 (%) is the percentual value of a chosen chemical compound quantity for F_HF95 NCO 300 formulation.

Relative quantity change Rel. Δ (%) was calculated using Equation (2):

(2) $$\mathrm{Rel}.∆\left(\%\right)=\frac{\left|∆\left(\%\right)\right|}{m_{\mathrm{rel}}\mathrm{NCO}\;150\;\left(\%\right)}\times 100$$

where |Δ (%)| is the absolute value of the calculated quantity change Δ (%), m_rel_ F_HF95 NCO 150 (%) is the corresponding percentual value of a chosen chemical compound quantity for F_HF95 NCO 150 formulation.

**Figure 3:**
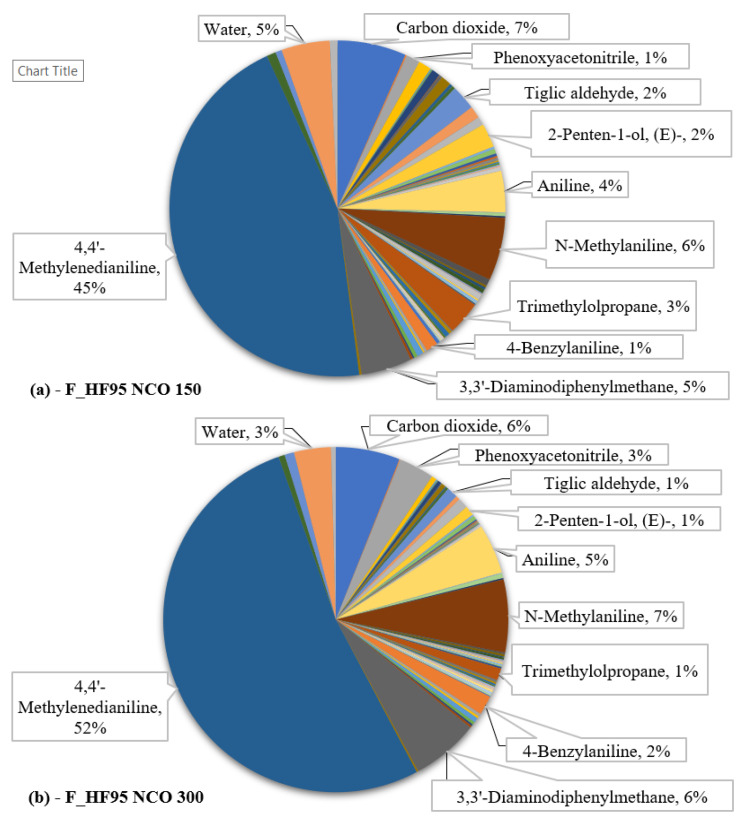
F_HF95 NCO 150 (**a**) and F_HF95 NCO 300 (**b**) compound fraction distribution according to the relative quantity values.

In order to better discern the differences in the chemical compound relative quantity values, Figure 3 shows the main thermal degradation product fraction parts out of the full obtained pyrolysis data for the F_HF95 NCO 150 and F_HF95 NCO 300 samples.

The released chemical compounds contain a mixture of fully oxidized products like CO_2_, partially oxidized products like CO and aldehydes and ketones, as well as aliphatic or aromatic hydrocarbons. According to the changes in the product relative quantities, the compounds that originate from the MDI remain in higher concentration as the NCO index is increased, whereas the compounds that originate from the polyol moiety have an observable decrease in concentration. The compounds with the highest quantified relative amount—4,4′-methylenedianiline (Rt = 38.071 min), N-methylaniline (Rt = 16.175 min), 3,3′-diaminodiphenylmethane (Rt = 35.723 min), aniline (Rt = 14.278 min), and 4-benzylaniline (Rt = 29.557 min)—are direct MDI thermal decomposition products, cumulatively 61.4% for H_FH95 NCO 150 and 72.0% for H_FH95 NCO 300. The total amount of the compounds that have been traced to have originated from the MDI are 70.1% for H_FH95 NCO 150 and 81.3% for H_FH95 NCO 300, accounting for approximately 2/3 (NCO 150) and 4/5 (NCO 300) of the total generated compound content. Pyrolysis products derived from catalysts like diethylene glycol (Rt = 15.264 min), cross-linkers such as trimethylolpropane (Rt = 23.019 min), and TOFA-based polyol thermal decomposition products like 1-hexadecanol (Rt = 31.573 min) are present.

The quantities of inorganic gases—CO_2_ (Rt = 5.287 min), CO (Rt = 24.167 min), H_2_O (vapor) (Rt = 39.136 min), NO (Rt = 38.503 min) and NO_2_ (Rt = 40.054 min)—apart from NH_3_ (Rt = 38.813 min), have reduced observably. The ammonia exception occurs because, during the thermal decomposition process, NH_3_ is essentially the penultimate product in the NCO-containing compound thermal decomposition chain, that is ultimately oxidized to one of the NO_x_ variety, the NO_2_:NO ratio depending on the available oxygen in the system. While acknowledging that CO_2_ and H_2_O can originate from other chemical ingredients used in the rigid PU-PIR foam production, these compounds are assumed to have originated mostly from the TOFA-based polyol during the thermal decomposition mid (T = 350–480 °C) and end (T ~ 600 °C) phases [26]. The observed trend for the relative amount of the generated inorganic gases and the sample NCO values is inversely proportional. This indicates that the added thermal stability to the rigid PU-PIR foam from the isocyanurate structures within the matrix is most likely responsible for the complication in the thermal decomposition processes by preventing the material from full decomposing under a high-temperature influence. Therefore, the excreted thermal decomposition products from the rigid PU-PIR foam with a higher NCO index will contain more of the volatile organic chemical compounds (VOCs) content-wise as can be seen from the Py-GC/MS results.

### 3.2. Functional Group Assessment for the Volatile Organic Compounds

The VOCs were also evaluated from their functional group aspect. The findings confirmed the presence of 38 alkene (of them 21 in benzene ring structure), 1 alkyne, 19 phenyl, 20 amine, 8 alcohol, 1 ether, 4 aldehyde, 2 ketone, 2 amide, 1 isocyanide, 1 isocyanate and 4 nitrile functional groups in the surveyed 63 organic chemical compounds. Chemical compound examples for each functional group are available in Table 5.

The majority (43) of the excreted compounds include a benzene ring structure somewhere within their structure, either possessing a phenyl group or being a substituted aromatic compound itself. Some of the identified compounds were even polycondensed aromatics and aromatic heterocyclic organic compounds, i.e., carbazole, well known for their effects on human health and the environment [28,29]. All these compounds have at least one GHS hazard code attributed to them from either the Health or Environmental hazard class.

A deeper insight in the properties of the identified thermal decomposition products, particularly the inherent danger to human health and the environment, was in order. Each compound was evaluated by the corresponding GHS hazard codes assigned to it from the physical, health, and environmental hazard classes (Table 6).

The results of the study are as follows: the identified chemical compounds possess 38 different hazard codes, i.e., eight physical hazard codes, 25 health hazards codes, and five environmental hazard codes. Twelve compounds have been assessed to be free of any assigned hazard codes at the time. Many of the evaluated chemical compounds possess more than one hazard code (phenyl isocyanate—a total of 12 hazard codes). The most common hazards in all the pyrolysis products are H315 “Causes skin irritation” (36%), H319 “Causes serious eye irritation” (28%), H335 “May cause respiratory irritation” (25%), and H302 “Harmful if swallowed” (23%). All the most commonly identified hazards are health hazards, and since upper respiratory tract irritants are believed to depend on the concentration alone [30], it makes the excreted chemical compounds innately harmful to human health. The chemical compound with the highest relative content value—4,4′-methylenedianiline (45.3% for PU and 52.4% for PIR)—is particularly dangerous, as it is one of the three identified compounds, that may cause cancer, and is also suspected of causing genetic defects. This chemical compound is found in abundance (Δ (%) ~ 55%) by Hiltz et al. [24] as well, where it is determined to be the primary thermal degradation product of the portion of the elastomer that incorporated MDI.

Overall, the results on the thermal degradation products that originate from the NCO moiety re-affirm the findings discovered by Stec and Hull regarding the chemical composition of fire gases of PU and PIR insulation materials [17]. Since both studied foams are nearly 100% organic materials, i.e., they consist of only carbon, oxygen, hydrogen, and nitrogen elements almost exclusively, the thermal degradation of the foams will produce a vast variety of chemical compounds, ranging from compounds formed primarily from the formulation ingredients like 4,4′-methylenedianiline from MDI to purely inorganic gases like CO_2_, NH_3_, and H_2_O. Comparing the obtained chemical compound compositions and quantities to the ones studied under under-ventilated or oxygen-poor conditions by Stec and Hull, H_FH95 NCO 150 produced almost twice (Rel. Δ (%) = 39.7%) the amount of the NO_2_ compared to H_FH95 NCO 300, similarly to the studied PU and PIR foam (Rel. Δ (%) = 51.3%) [17]. It happens due to the lower NCO content of the H_FH95 NCO 150, which would otherwise have inhibited the propagation of thermal degradation end products.

Due to the lack of analytical pyrolysis studies of renewable source-based PU and PIR materials, the chemical compound quantity comparison is not suitable to evaluate the effect of the TOFA-based polyol thermal degradation products on the overall chemical compound composition. Only the chemical compounds that originate from the pMDI can be objectively compared to other study results, specifically the studies that employ similar methodology for the chemical compound analysis. However, various thermal degradation studies where the conventionally produced (oil-based) polyols are replaced with renewable source-based polyols [1,4,5,8,31] show that these formulations possess distinctive thermal degradation trends. Therefore, it is reasonable to assume that the renewable source-based polyol has equally diminished the MDI thermal degradation product quantities during the initial thermal degradation stages (T from ~210 °C to ~230 °C) and facilitated the thermal degradation of the MDI derivatized products in the end-point pyrolytic thermal degradation stage (T from ~600 °C to ~650 °C), and this assumption is supported by conclusions made in a previous study on the rigid PU and PIR foam thermal degradation [26], that included the analysis on the formulations studied in this study, too. For these reasons, the presence of the renewable source-based polyols in the foam is beneficial for the insulation properties of the material because the diminishing of MDI thermal degradation product quantities equals increased thermal stability, and the additional oxygen from the polyol matrix allows for a greater output of inorganic gases like CO_2_ and H_2_O over VOCs.

## 4. Conclusions

In the study, two high-functionality tall-oil polyol based rigid polyurethane (PU) and polyisocyanurate (PIR) foam formulations with isocyanate (NCO) indexes 150 and 300 were obtained and thermal degradation products of them were studied by analytical pyrolysis (Py-GC/MS). The results show that the main pyrolysates originated from the decomposition of the isocyanate moiety part of the rigid PU-PIR foam, i.e., 4,4′-diphenylmethane diisocyanate (MDI), primarily forming 4,4′-methylenedianiline, 3,3′-diaminodiphenylmethane, N-methylaniline, aniline, 4-benzylaniline and phenyl isocyanate. The amount of certain chemical components increased by up to 29% when measured across the samples (NCO indexes 150–300). The majority (43 out of 69) of the excreted compounds included a benzene ring structure somewhere within their structure, either possessing a phenyl group or being a substituted aromatic compound itself. The total amount of the chemical compounds traced to have originated from the MDI was 70.1% for PUR foams NCO index 150 and 81.3% for PIR foams NCO index 300 of the total generated compound volume.

With nearly 4/5 of the pyrolysates originating from the isocyanate moiety, it can be concluded that the smoke from a bio-based rigid PU-PIR foam, where the foam contains no additives like flame retardants or smoke suppressants, could pose a potential threat to human health and the environment. Therefore, when studying either fully bio-based or partially bio-based materials, it is suggested to evaluate if the positive gains of including more bio-based material in non-renewable products during the material production stage outweigh the potential negative effects on health and the environment. This step should be imperative if the product could be potentially exposed to conditions unsuitable for maintaining its chemical and structural integrity.

A thorough pyrolysate hazard evaluation process led to the observation that most of the identified threats were hazards related to health. Therefore, when studying the rigid PU-PIR foam thermal degradation behavior or assessing the properties related to thermal stability, the use of appropriate personal safety equipment like respirators with chemical cartridges containing at least volatile organic compound (VOC) sorbents is strongly recommended. The respirator canisters ought to contain multiple protective sorbent types, because the released chemical compounds are highly varied, i.e., VOCs, inorganic gases (including CO, NO and NO_2_), ammonia and its organic derivatives. In addition to the use of respiratory protective equipment when assessing the thermal degradation behavior and properties of PU and PIR foams, a proper local exhaust ventilation should also be considered.

## Figures and Tables

**Table 1:** Materials used in the formulations.

Chemical Component	Structure
TOFA-based polyol [27], derived from: (1) oleic acid; (2) linoleic acid	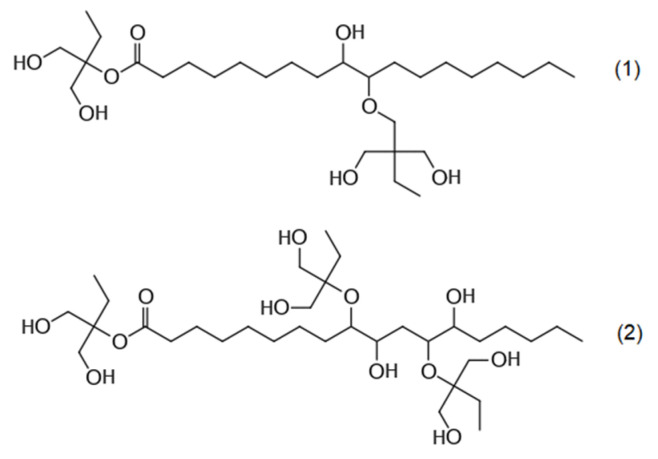
Lupranol^®^ 3422 (sorbitol-based polyether polyol) (BASF, Ludwigshafen, Germany)	(The exact chemical structure has not been disclosed)
Solkane^®^ 365/277 (86–92% of 1,1,1,3,3 pentafluorobutane; 8–14% of 1,1,1,2,3,3,3-heptafluoropropane) (Solvay Special Chemicals, Düsseldorf, Germany)	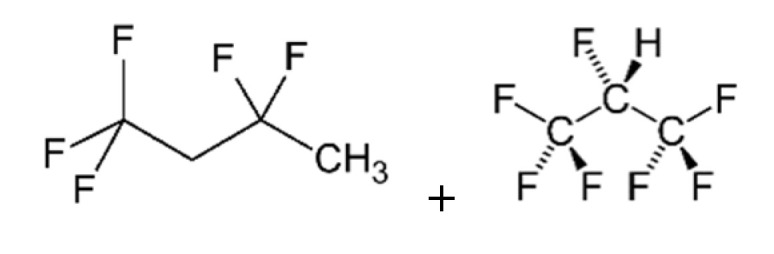
Water (deionized)	H_2_O
Tertiary amine (Polycat^®^ 5) N,N,N′,N′′,N′′-pentamethyldiethylenetriamine (PMDTA) (Evonik Industries, Essen, Germany)	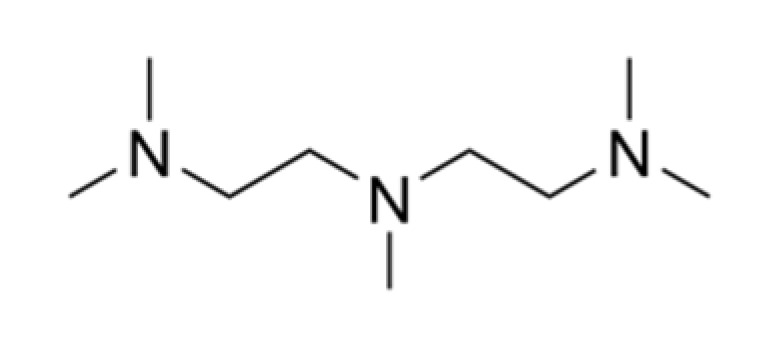
30% potassium acetate in DEG (PC CAT TKA 30 and PC CAT Q 7-2) catalysts (Air Products Europe Chemicals B.V., Utrecht, The Netherlands)	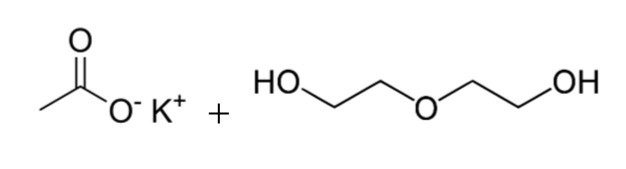
Niax Silicone L-6915 (Momentive Performance Materials Inc., Geesthacht, Germany)	(The exact chemical structure has not been disclosed)
Desmodur 44V20L (Covestro, Leverkusen, Germany; NCO content is 30.5–32.5 wt%)	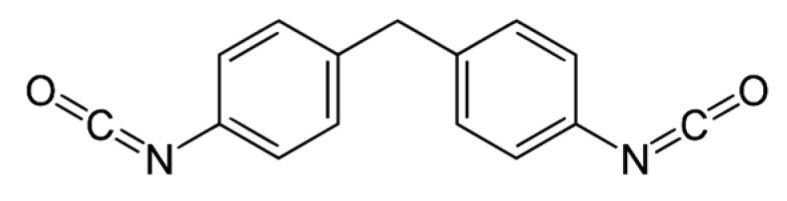

**Table 2:** Formulation additives in the rigid PU-PIR foams.

Polyol Formulation (pbw)		
Sample Code *	F_HF95 NCO 150	F_HF95 NCO 300
NCO index	150	300
TOFA-based polyol	95.0	95.0
Lupranol 3422	5.0	5.0
Blowing reagent (water)	0.5	0.5
Blowing agent (Solkane 365/277)	40.0	70.0
Catalyst (Polycat 5)	2.0	2.0
Catalyst (PC CAT TKA 30)	1.5	2.5
Catalyst (PC CAT Q 7-2)	2.0	2.0
Surfactant (L-6915)	2.0	2.0
pMDI (Desmodur 44V20L)	150.0	299.9

* Sample codes are retained as in articles [10,26].

**Table 3:** Characteristics of F_HF95 samples (NCO 150 and 300).

Characteristic	F_HF95 NCO 150	F_HF95 NCO 300
Start time, s	20	20
String time, s	37	75
Tack free time, s	50	125
Temperature of polyol component before foaming, °C	24	24
Apparent density of molded PU-PIR foams, kg/m^3^	31.6	35.6
Closed cell content, %	93	96
Mc, g/mol	499	549
Polyol moiety viscosity, mPa∙s	62,560 ± 40	62,560 ± 40

**Table 4:** Identification of the chromatographic peaks of F_HF95 samples (NCO 150 and 300).

No.	Peak Identification	Rt (Min)	Mrel NCO 150 (%)	Mrel NCO 300 (%)	Δ (%)	Rel. Δ (%)	GHS Hazard Statements
1	Carbon dioxide	5.287	6.65	6.02	−0.63	−9.5	H280; H281
2	1-Butene	5.405	0.14	0.09	−0.05	−35.7	H220; H221; H280
3	Phenoxyacetonitrile	5.474	1.34	3.32	1.98	147.8	H302; H312; H332
4	cis-1,2-Dimethylcyclopropane	5.575	1.25	0.49	−0.76	−60.8	Not classified
5	Isoprene	5.633	0.10	0.12	0.02	20.0	H224; H341; H350; H412
6	3-Penten-1-yne	5.826	0.11	0.07	−0.04	−36.4	Not classified
7	1-Hexene	5.898	0.82	0.37	−0.45	−54.9	H225; H304; H319
8	Methyl isocyanide	6.120	0.06	trace	−	−	H302; H312; H332; H373
9	2-Butenal	6.218	0.27	0.09	−0.18	−66.7	H225; H301; H310; H311; H315; H318; H330; H335; H341; H373; H400
10	1-Heptene	6.524	0.99	0.40	−0.59	−59.6	H225; H304; H400; H410
11	1,2,5-Hexatriene	6.641	0.30	0.12	−0.18	−60.0	Not classified
12	Benzene	6.767	0.32	0.21	−0.11	−34.4	H225; H304; H315; H319; H340; H350; H372
13	Tiglic aldehyde	7.063	2.39	1.03	−1.36	−56.9	H225; H315; H319; H335; H411
14	1-Octene	7.583	1.16	0.40	−0.76	−65.5	H225; H304; H315; H400; H410; H411
15	Toluene	8.029	0.81	0.96	0.15	18.5	H225; H304; H315; H336; H361d; H373
16	2-Penten-1-ol, (E)-	8.656	2.32	0.91	−1.41	−60.8	H226
17	M-Xylene	9.581	0.23	0.16	−0.07	−30.4	H226; H304; H312; H315; H318; H332
18	P-Xylene	9.736	0.45	0.37	−0.08	−17.8	H226; H304; H312; H315; H319; H332; H335
19	O-Xylene	10.325	0.26	0.13	−0.13	−50.0	H225; H226; H304; H312; H315; H319; H332; H335
20	Styrene	10.489	0.17	0.09	−0.08	−47.1	H226; H315; H319; H332; H361d; H372
21	2-Heptanone	10.859	0.27	0.11	−0.16	−59.3	H226; H302; H332
22	1-Decene	11.023	0.14	0.07	−0.07	−50.0	H226; H304; H400; H410
23	Propylbenzene	11.367	0.11	0.06	−0.05	−45.5	H226; H304; H335; H411
24	3-Ethyltoluene	11.564	0.17	0.06	−0.11	−64.7	H226; H304; H336; H411
25	1-Dodecene	13.094	0.11	0.07	−0.04	−36.4	H304; H315
26	Butylbenzene	13.509	0.12	0.04	−0.08	−66.7	H226; H315; H319; H400; H410
27	2-Ethylhexanol	13.864	0.38	0.18	−0.20	−52.6	H315; H319; H332; H335
28	Aniline	14.278	3.99	4.68	0.69	17.3	H301; H311; H317; H318; H331; H341; H351; H372; H400
29	Benzonitrile	14.439	0.05	0.07	0.02	40.0	H302; H312
30	Diethylene glycol	15.264	0.31	0.44	0.13	41.9	H302
31	Pentylbenzene	15.660	0.15	0.13	−0.02	−13.3	H411; H412
32	N-Methylaniline	16.175	6.08	6.83	0.75	12.3	H301; H311; H331; H373; H400; H410
33	2-Ethyl-2-methyl-1,3-propanediol	16.708	0.64	0.16	−0.48	−75.0	Not classified
34	Benzyl cyanide	17.129	0.21	0.22	0.01	4.8	H301; H302; H311; H330
35	N-Methyl-m-toluidine	17.554	0.22	0.09	−0.13	−59.1	H301; H311 H331; H373; H412
36	2,5-Dimethylaniline	17.925	0.39	0.20	−0.19	−48.7	H301; H311; H331; H373; H411
37	2-Ethylaniline	18.033	0.11	0.10	−0.01	−9.1	H301; H302; H311; H319; H330; H331; H373;
38	cis-3-Tetradecene	19.166	0.05	0.06	0.01	20.0	Not classified
39	Carbazole	19.606	0.55	0.15	−0.40	−72.7	H315; H341; H351; H400; H411; H413
40	4-Vinylaniline	20.090	0.16	0.11	−0.05	−31.3	H302; H312; H315; H319; H332; H334; H335; H351; H373
41	7-Methylquinoline	21.750	0.29	0.17	−0.12	−41.4	H315; H318; H319; H335
42	Indolizine	22.136	0.05	0.10	0.05	100.0	H315; H319
43	5H-Cyclopenta[b]pyridine	22.431	0.10	0.16	0.06	60.0	Not classified
44	Trimethylolpropane	23.019	3.26	1.12	−2.14	−65.6	Not classified
45	4-Aminophenylacetonitrile	23.831	0.11	0.22	0.11	100.0	H302; H312; H315; H319; H332; H335
46	2,8-Dimethylquinoline	24.018	0.31	0.17	−0.14	−45.2	H302; H315; H318; H335
47	Carbon monoxide	24.167	0.44	0.16	−0.28	−63.6	H220; H331; H360d; H372
48	8-Propylquinoline	25.657	0.19	0.11	−0.08	−42.1	Not classified
49	1-Octadecene	25.975	0.00	0.14	0.14	140.0	H304
50	Oxindole	26.306	0.10	0.12	0.02	20.0	Not classified
51	N-Methylacetanilide	26.633	0.18	0.09	−0.09	−50.0	H301
52	Benzimidazole	26.922	0.09	0.21	0.12	133.3	H302; H315; H319; H335
53	N-Formylindoline	27.870	0.10	0.22	0.12	120.0	Not classified
54	N-Methyldiphenylamine	28.322	0.07	0.15	0.08	114.3	H315; H319; H335
55	9-Octadecanone	28.581	0.32	0.08	−0.24	−75.0	Not classified
56	4-Benzylaniline	29.557	1.17	1.79	0.62	53.0	H302; H312; H315; H319; H332; H335;
57	Acridine	29.992	0.26	0.28	0.02	7.7	H302; H315; H319; H335;
58	Acrolein	30.767	0.14	0.21	0.07	50.0	H225; H300; H311; H314; H330; H400; H410
59	Phenol	31.035	0.63	0.46	−0.17	−27.0	H301; H311; H314; H331; H341; H373
60	1-Hexadecanol	31.573	0.34	0.26	−0.08	−23.5	H315; H319; H335; H400; H410; H411; H412; H413
61	9-Vinylcarbazole	31.874	0.14	0.16	0.02	14.3	H302; H312; H315; H317; H341; H400; H410
62	Phenyl isocyanate	32.398	0.25	0.20	−0.05	−20.0	H226; H302; H314; H317; H318; H330; H334; H335; H400; H410; H411; H412
63	3,3′-Diaminodiphenylmethane	35.723	4.89	6.31	1.42	29.0	H302; H312; H315; H319; H332; H335; H351
64	Methanol	36.973	0.22	0.16	−0.06	−27.3	H225; H301; H311; H331; H370
65	4,4′-Methylenedianiline	38.071	45.26	52.41	7.15	15.8	H317; H341; H350; H370; H373; H411
66	Nitric oxide	38.503	0.89	0.58	−0.31	−34.8	H270; H280; H314; H318; H330; H331; H373
67	Ammonia	38.813	0.61	0.89	0.28	45.9	H221; H314; H331; H400
68	Water	39.136	4.67	3.46	−1.21	−25.9	Not classified
69	Nitrogen dioxide	40.054	0.73	0.44	−0.29	−39.7	H270; H280; H314; H318; H330

**Table 5:** Functional group assessment in the rigid PU-PIR foam thermal decomposition products.

Functional Group/Bond	Counts	Examples
Alkene	38	1-Butene, Isoprene, 1-Hexene, 1-Dodecene
Alkene incorporated in benzene ring structure	21	M-Xylene, P-Xylene, O-Xylene, 3-Ethyltoluene, 9-Vinylcarbazole, Carbazole
Alkyne	1	3-Penten-1-yne
Phenyl	19	4,4′-Methylenedianiline, N-Methyldiphenylamine
Amine	20	Aniline, N-Methylaniline, 2,5-Dimethylaniline
Alcohol	8	2-Ethylhexanol, Trimethylolpropane, Diethylene glycol
Ether	1	Diethylene glycol
Aldehyde	4	2-Butenal, Tiglic aldehyde, N-Formylindoline, Acrolein
Ketone	2	2-Heptanone, 9-Octadecanone
Amide	2	N-Methylacetanilide, Oxindole
Isocyanide	1	Methyl isocyanide
Isocyanate	1	Phenyl isocyanate
Nitrile	4	Benzyl cyanide, Benzonitrile, Phenoxyacetonitrile

**Table 6:** Evaluation of the rigid PU-PIR foam thermal decomposition product hazards.

GHS Hazard	H Code	Phrase	Compounds with H Code	Fraction of all Pyrolysis Products (%)
Physical hazards	H220	Extremely flammable gas	2	3
	H221	Flammable gas	2	3
	H224	Extremely flammable liquid and vapor	1	1
	H225	Highly flammable liquid and vapor	10	14
	H226	Flammable liquid and vapor	11	16
	H270	May cause or intensify fire; oxidizer	2	3
	H280	Contains gas under pressure; may explode if heated	4	6
	H281	Contains refrigerated gas; may cause cryogenic burns or injury	1	1
Health hazards	H301	Toxic if swallowed	10	14
	H302	Harmful if swallowed	16	23
	H304	May be fatal if swallowed and enters airways	13	19
	H310	Fatal in contact with skin	1	1
	H311	Toxic in contact with skin	10	14
	H312	Harmful in contact with skin	11	16
	H314	Causes severe skin burns and eye damage	6	9
	H315	Causes skin irritation	25	36
	H317	May cause an allergic skin reaction	4	6
	H318	Causes serious eye damage	8	12
	H319	Causes serious eye irritation	19	28
	H330	Fatal if inhaled	7	10
	H331	Toxic if inhaled	10	14
	H332	Harmful if inhaled	12	17
	H334	May cause allergy or asthma symptoms or breathing difficulties if inhaled	2	3
	H335	May cause respiratory irritation	17	25
	H336	May cause drowsiness or dizziness	2	3
	H340	May cause genetic defects	1	1
	H341	Suspected of causing genetic defects	7	10
	H350	May cause cancer	3	4
	H351	Suspected of causing cancer	4	6
	H361d	Suspected of damaging the unborn child	2	3
	H370	Causes damage to organs	2	3
	H372	Causes damage to organs through prolonged or repeated exposure	4	6
	H373	May cause damage to organs through prolonged or repeated exposure	11	16
Environmental hazards	H400	Very toxic to aquatic life	13	19
	H410	Very toxic to aquatic life with long-lasting effects	9	13
	H411	Toxic to aquatic life with long-lasting effects	10	14
	H412	Harmful to aquatic life with long-lasting effects	5	7
	H413	May cause long-lasting harmful effects to aquatic life	2	3
Not classified	-	-	12	17

## Data Availability

Data is contained within this article.

## References

[1] Prociak A., Kurańska M., Cabulis U., Kirpluks M. (2017). Rapeseed oil as main component in synthesis of bio-polyurethane-polyisocyanurate porous materials modified with carbon fibers. Polym. Test..

[2] Kirpluks M., Kalnbunde D., Walterova Z., Cabulis U. (2017). Rapeseed Oil as Feedstock for High Functionality Polyol Synthesis. J. Renew. Mater..

[3] Kurańska M., Prociak A., Cabulis U., Kirpluks M., Ryszkowska J., Auguścik M. (2017). Innovative porous polyurethane-polyisocyanurate foams based on rapeseed oil and modified with expandable graphite. Ind. Crop. Prod..

[4] Fridrihsone-Girone A., Stirna U. (2014). Characterization of polyurethane networks based on rapeseed oil derived polyol. Polimery.

[5] Pietrzak K., Kirpluks M., Cabulis U., Ryszkowska J. (2014). Effect of the addition of tall oil-based polyols on the thermal and mechanical properties of ureaurethane elastomers. Polym. Degrad. Stab..

[6] Kurańska M., Prociak A., Cabulis U., Kirpluks M. (2015). Water-blown polyurethane-polyisocyanurate foams based on bio-polyols with wood fibers. Polimery.

[7] Kurańska M., Prociak A., Kirpluks M., Cabulis U. (2015). Polyurethane-polyisocyanurate foams modified with hydroxyl derivatives of rapeseed oil. Ind. Crop. Prod..

[8] Kurańska M., Cabulis U., Auguścik M., Prociak A., Ryszkowska J., Kirpluks M. (2016). Bio-based polyurethane-polyisocyanurate composites with an intumescent flame retardant. Polym. Degrad. Stab..

[9] Javni I., Zhang W., Petrović Z.S. (2004). Soybean-oil-based polyisocyanurate rigid foams. J. Polym. Environ..

[10] Reinerte S., Kirpluks M., Cabulis U. (2019). Thermal degradation of highly crosslinked rigid PU-PIR foams based on high functionality tall oil polyol. Polym. Degrad. Stab..

[11] Gadhave R.V., Kasbe P.S., Mahanwar P.A., Gadekar P.T. (2019). Synthesis and characterization of lignin-polyurethane based wood adhesive. Int. J. Adhes. Adhes..

[12] García J.L., Pans G., Phanopoulos C. (2018). Use of lignin in polyurethane-based structural wood adhesives. J. Adhes..

[13] Aristri M.A., Lubis M.A.R., Yadav S.M., Antov P., Papadopoulos A.N., Pizzi A., Fatriasari W., Ismayati M., Iswanto A.H. (2021). Recent Developments in Lignin- and Tannin-Based Non-Isocyanate Polyurethane Resins for Wood Adhesives—A Review. Appl. Sci..

[14] Tavares L.B., Boas C.V., Schleder G.R., Nacas A.M., Rosa D.S., Santos D.J. (2016). Bio-based polyurethane prepared from Kraft lignin and modified castor oil. Express Polym. Lett..

[15] Gama N.V., Ferreira A., Barros-Timmons A. (2018). Polyurethane foams: Past, present, and future. Materials.

[16] Kaplan H.L., Grand A.F., Hartzell G.E. (1984). Toxicity and the smoke problem. Fire Saf. J..

[17] Stec A.A., Hull T.R. (2011). Assessment of the fire toxicity of building insulation materials. Energy Build..

[18] McKenna S.T., Hull T.R. (2016). The fire toxicity of polyurethane foams. Fire Sci. Rev..

[19] Wang B., Sheng H., Shi Y., Song L., Zhang Y., Hu Y., Hu W. (2016). The influence of zinc hydroxystannate on reducing toxic gases (CO, NO_x_ and HCN) generation and fire hazards of thermoplastic polyurethane composites. J. Hazard. Mater..

[20] Yuan Y., Wang W., Shi Y., Song L., Ma C., Hu Y. (2019). The influence of highly dispersed Cu_2_O-anchored MoS_2_ hybrids on reducing smoke toxicity and fire hazards for rigid polyurethane foam. J. Hazard. Mater..

[21] Xu W., Wang G., Xu J., Liu Y., Chen R., Yan H. (2019). Modification of diatomite with melamine coated zeolitic imidazolate framework-8 as an effective flame retardant to enhance flame retardancy and smoke suppression of rigid polyurethane foam. J. Hazard. Mater..

[22] Li M.E., Wang S.H., Han L.X., Yuan W.J., Cheng J.B., Zhang A.N., Zhao H.B., Wang Y.Z. (2019). Hierarchically porous SiO_2_/polyurethane foam composites towards excellent thermal insulating, flame-retardant and smoke-suppressant performances. J. Hazard. Mater..

[23] Wi S., Berardi U., Di Loreto S., Kim S. (2020). Microstructure and thermal characterization of aerogel–graphite polyurethane spray-foam composite for high efficiency thermal energy utilization. J. Hazard. Mater..

[24] Hiltz J.A. (2015). Analytical pyrolysis gas chromatography/mass spectrometry (py-GC/MS) of poly(ether urethane)s, poly(ether urea)s and poly(ether urethane-urea)s. J. Anal. Appl. Pyrolysis.

[25] European Commission (2018). Commission Regulation (EU) 2018/669 of 16 April 2018 amending, for the purposes of its adaptation to technical and scientific progress, Regulation (EC) No 1272/2008 of the European Parliament and of the Council on classification, labelling and packaging of substances and mixtures. OJ.

[26] Reinerte S., Avotina L., Zarins A., Cabulis U., Viksna A. (2020). TG/DTA-FTIR as a method for analysis of tall oil based rigid polyurethane foam decomposition gaseous products in a low oxygen environment. Polym. Degrad. Stab..

[27] Kirpluks M., Vanags E., Abolins A., Michalowski S., Fridrihsone A., Cabulis U. (2020). High Functionality Bio-Polyols from Tall Oil and Rigid Polyurethane Foams Formulated Solely Using Bio-Polyols. Materials.

[28] Kim K.H., Jahan S.A., Kabir E., Brown R.C.J. (2013). A review of airborne polycyclic aromatic hydrocarbons (PAHs) and their human health effects. Environ. Int..

[29] Mumbo J., Henkelmann B., Abdelaziz A., Pfister G., Nguyen N., Schroll R., Munch J.C., Schramm K.W. (2015). Persistence and dioxin-like toxicity of carbazole and chlorocarbazoles in soil. Environ. Sci. Pollut. Res..

[30] Purser D.A. The Application of Exposure Concentration and Dose to Evaluation of Effects of Irritants as Components of Fire Hazard. Proceedings of the Interflam Conference Proceedings.

[31] Hejna A., Kirpluks M., Kosmela P., Cabulis U., Haponiuk J., Piszczyk Ł. (2017). The influence of crude glycerol and castor oil-based polyol on the structure and performance of rigid polyurethane-polyisocyanurate foams. Ind. Crop. Prod..

